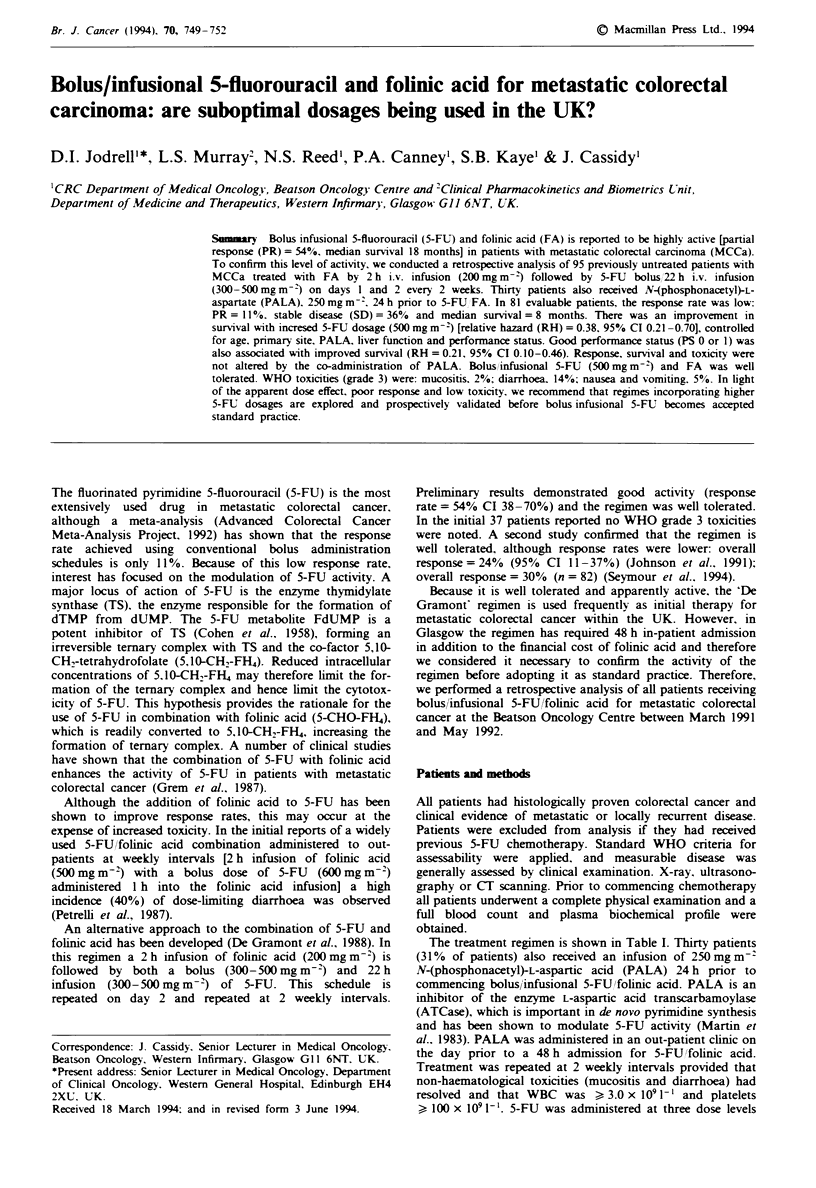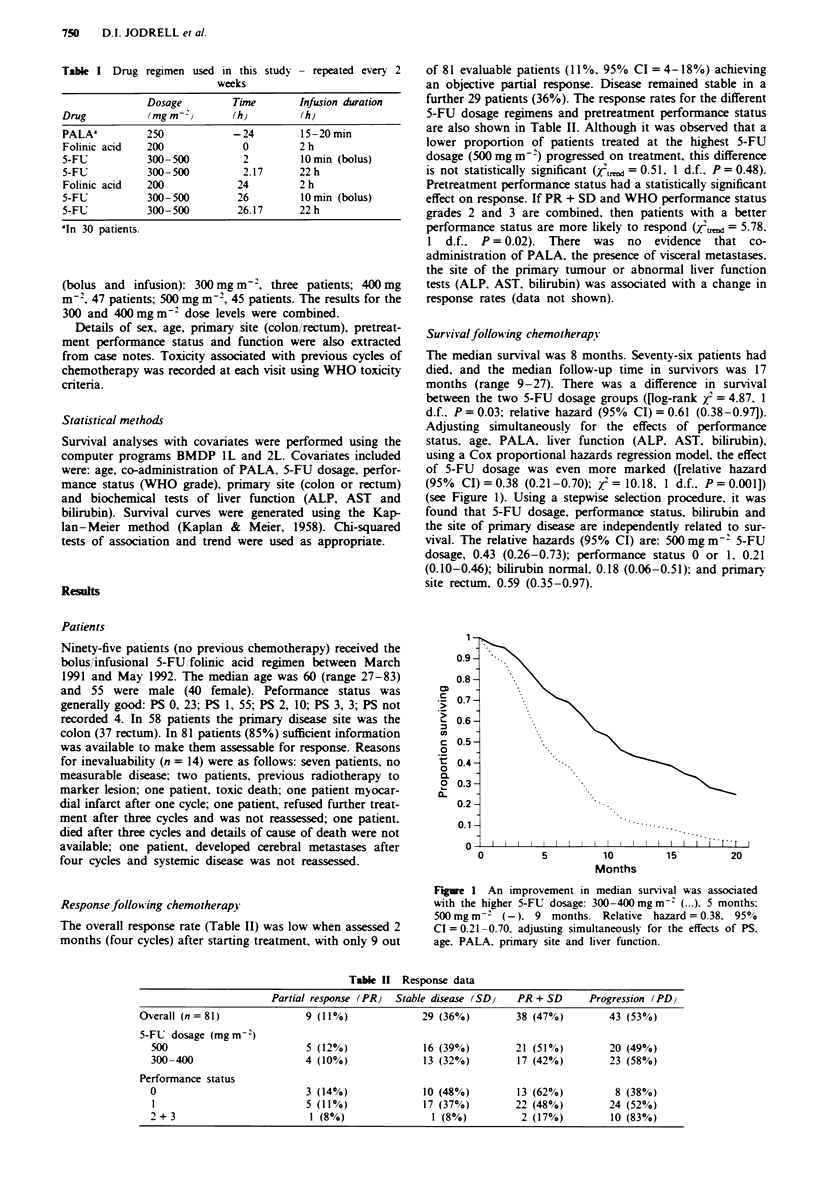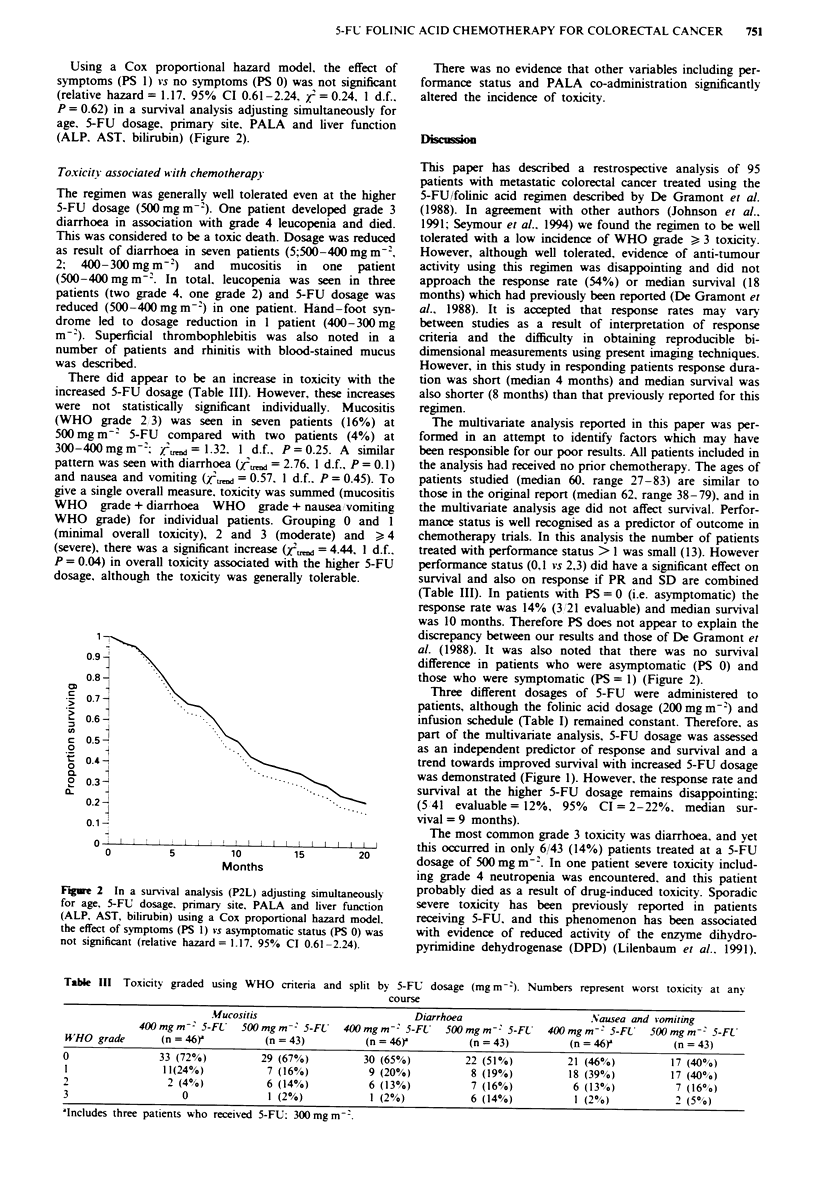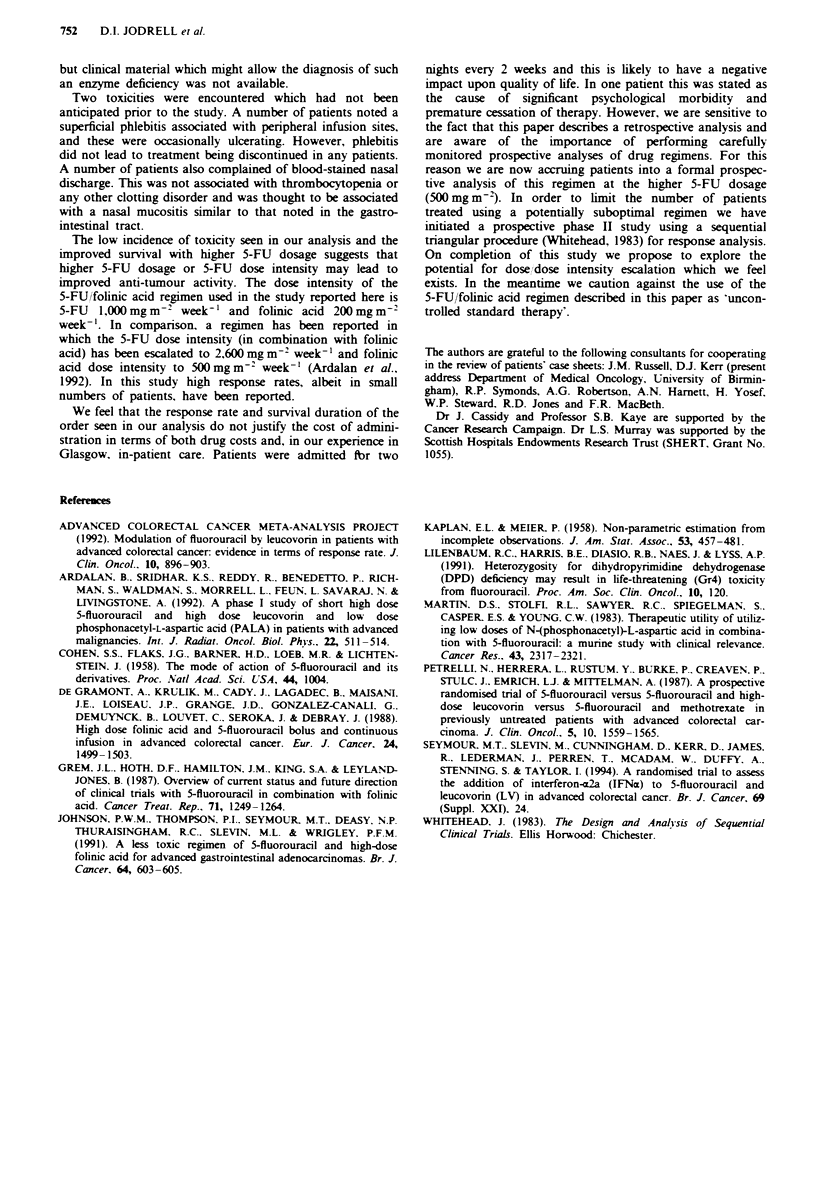# Bolus/infusional 5-fluorouracil and folinic acid for metastatic colorectal carcinoma: are suboptimal dosages being used in the UK?

**DOI:** 10.1038/bjc.1994.389

**Published:** 1994-10

**Authors:** D. I. Jodrell, L. S. Murray, N. S. Reed, P. A. Canney, S. B. Kaye, J. Cassidy

**Affiliations:** CRC Department of Medical Oncology, Beatson Oncology Centre, Glasgow, UK.

## Abstract

Bolus/infusional 5-fluorouracil (5-FU) and folinic acid (FA) is reported to be highly active [partial response (PR) = 54%, median survival 18 months] in patients with metastatic colorectal carcinoma (MCCa). To confirm this level of activity, we conducted a retrospective analysis of 95 previously untreated patients with MCCa treated with FA by 2 h i.v. infusion (200 mg m-2) followed by 5-FU bolus/22 h i.v. infusion (300-500 mg m-2) on days 1 and 2 every 2 weeks. Thirty patients also received N-(phosphonacetyl)-L-aspartate (PALA), 250 mg m-2, 24 h prior to 5-FU/FA. In 81 evaluable patients, the response rate was low: PR = 11%, stable disease (SD) = 36% and median survival = 8 months. There was an improvement in survival with increased 5-FU dosage (500 mg m-2) [relative hazard (RH) = 0.38, 95% CI 0.21-0.70], controlled for age, primary site, PALA, liver function and performance status. Good performance status (PS 0 or 1) was also associated with improved survival (RH = 0.21, 95% CI 0.10-0.46). Response, survival and toxicity were not altered by the co-administration of PALA. Bolus/infusional 5-FU (500 mg m-2) and FA was well tolerated. WHO toxicities (grade 3) were: mucositis, 2%; diarrhoea, 14%; nausea and vomiting, 5%. In light of the apparent dose effect, poor response and low toxicity, we recommend that regimes incorporating higher 5-FU dosages are explored and prospectively validated before bolus/infusional 5-FU becomes accepted standard practice.


Br. J. Cancer (1994), 70, 749-752
© Macmillan Press Ltd., 1994

### Bolus/infusional 5-fluorouracil and folinic acid for metastatic colorectal carcinoma: are suboptimal dosages being used in the UK?

D.I. Jodrell[1]*, L.S. Murray[2], N.S. Reed[1], P.A. Canney[1], S.B. Kaye[1] & J. Cassidy[1]

[1]*CRC Department of Medical Oncology, Beatson Oncology Centre and* [2]*Clinical Pharmacokinetics and Biometrics Unit, Department of Medicine and Therapeutics, Western Infirmary, Glasgow G11 6NT, UK.*

**Summary** Bolus infusional 5-fluorouracil (5-FU) and folinic acid (FA) is reported to be highly active [partial response (PR) = 54%, median survival 18 months] in patients with metastatic colorectal carcinoma (MCCa). To confirm this level of activity, we conducted a retrospective analysis of 95 previously untreated patients with MCCa treated with FA by 2 h i.v. infusion (200 mg $m^{-2}$) followed by 5-FU bolus/22 h i.v. infusion (300-500 mg $m^{-2}$) on days 1 and 2 every 2 weeks. Thirty patients also received *N*-(phosphonacetyl)-L-aspartate (PALA), 250 mg $m^{-2}$, 24 h prior to 5-FU/FA. In 81 evaluable patients, the response rate was low: PR = 11%, stable disease (SD) = 36% and median survival = 8 months. There was an improvement in survival with incresed 5-FU dosage (500 mg $m^{-2}$) [relative hazard (RH) = 0.38, 95% CI 0.21-0.70], controlled for age, primary site, PALA, liver function and performance status. Good performance status (PS 0 or 1) was also associated with improved survival (RH = 0.21, 95% CI 0.10-0.46). Response, survival and toxicity were not altered by the co-administration of PALA. Bolus/infusional 5-FU (500 mg $m^{-2}$) and FA was well tolerated. WHO toxicities (grade 3) were: mucositis, 2%; diarrhoea, 14%; nausea and vomiting, 5%. In light of the apparent dose effect, poor response and low toxicity, we recommend that regimes incorporating higher 5-FU dosages are explored and prospectively validated before bolus infusional 5-FU becomes accepted standard practice.

The fluorinated pyrimidine 5-fluorouracil (5-FU) is the most extensively used drug in metastatic colorectal cancer, although a meta-analysis (Advanced Colorectal Cancer Meta-Analysis Project, 1992) has shown that the response rate achieved using conventional bolus administration schedules is only 11%. Because of this low response rate, interest has focused on the modulation of 5-FU activity. A major locus of action of 5-FU is the enzyme thymidylate synthase (TS), the enzyme responsible for the formation of dTMP from dUMP. The 5-FU metabolite FdUMP is a potent inhibitor of TS (Cohen *et al.*, 1958), forming an irreversible ternary complex with TS and the co-factor 5,10-$CH_2$-tetrahydrofolate (5,10-$CH_2$-$FH_4$). Reduced intracellular concentrations of 5,10-$CH_2$-$FH_4$ may therefore limit the formation of the ternary complex and hence limit the cytotoxicity of 5-FU. This hypothesis provides the rationale for the use of 5-FU in combination with folinic acid (5-CHO-$FH_4$), which is readily converted to 5,10-$CH_2$-$FH_4$, increasing the formation of ternary complex. A number of clinical studies have shown that the combination of 5-FU with folinic acid enhances the activity of 5-FU in patients with metastatic colorectal cancer (Grem *et al.*, 1987).

Although the addition of folinic acid to 5-FU has been shown to improve response rates, this may occur at the expense of increased toxicity. In the initial reports of a widely used 5-FU/folinic acid combination administered to out-patients at weekly intervals [2 h infusion of folinic acid (500 mg $m^{-2}$) with a bolus dose of 5-FU (600 mg $m^{-2}$) administered 1 h into the folinic acid infusion] a high incidence (40%) of dose-limiting diarrhoea was observed (Petrelli *et al.*, 1987).

An alternative approach to the combination of 5-FU and folinic acid has been developed (De Gramont *et al.*, 1988). In this regimen a 2 h infusion of folinic acid (200 mg $m^{-2}$) is followed by both a bolus (300-500 mg $m^{-2}$) and 22 h infusion (300-500 mg $m^{-2}$) of 5-FU. This schedule is repeated on day 2 and repeated at 2 weekly intervals. Preliminary results demonstrated good activity (response rate = 54% CI 38-70%) and the regimen was well tolerated. In the initial 37 patients reported no WHO grade 3 toxicities were noted. A second study confirmed that the regimen is well tolerated, although response rates were lower: overall response = 24% (95% CI 11-37%) (Johnson *et al.*, 1991); overall response = 30% (*n* = 82) (Seymour *et al.*, 1994).

Because it is well tolerated and apparently active, the 'De Gramont' regimen is used frequently as initial therapy for metastatic colorectal cancer within the UK. However, in Glasgow the regimen has required 48 h in-patient admission in addition to the financial cost of folinic acid and therefore we considered it necessary to confirm the activity of the regimen before adopting it as standard practice. Therefore, we performed a retrospective analysis of all patients receiving bolus/infusional 5-FU/folinic acid for metastatic colorectal cancer at the Beatson Oncology Centre between March 1991 and May 1992.

### Patients and methods

All patients had histologically proven colorectal cancer and clinical evidence of metastatic or locally recurrent disease. Patients were excluded from analysis if they had received previous 5-FU chemotherapy. Standard WHO criteria for assessability were applied, and measurable disease was generally assessed by clinical examination, X-ray, ultrasonography or CT scanning. Prior to commencing chemotherapy all patients underwent a complete physical examination and a full blood count and plasma biochemical profile were obtained.

The treatment regimen is shown in Table I. Thirty patients (31% of patients) also received an infusion of 250 mg $m^{-2}$ *N*-(phosphonacetyl)-L-aspartic acid (PALA) 24 h prior to commencing bolus/infusional 5-FU/folinic acid. PALA is an inhibitor of the enzyme L-aspartic acid transcarbamoylase (ATCase), which is important in *de novo* pyrimidine synthesis and has been shown to modulate 5-FU activity (Martin *et al.*, 1983). PALA was administered in an out-patient clinic on the day prior to a 48 h admission for 5-FU/folinic acid. Treatment was repeated at 2 weekly intervals provided that non-haematological toxicities (mucositis and diarrhoea) had resolved and that WBC was $\geqslant 3.0 \times 10^9 l^{-1}$ and platelets $\geqslant 100 \times 10^9 l^{-1}$. 5-FU was administered at three dose levels

Correspondence: J. Cassidy, Senior Lecturer in Medical Oncology, Beatson Oncology, Western Infirmary, Glasgow G11 6NT, UK.
*Present address: Senior Lecturer in Medical Oncology, Department of Clinical Oncology, Western General Hospital, Edinburgh EH4 2XU, UK.
Received 18 March 1994; and in revised form 3 June 1994.

**Table I** Drug regimen used in this study – repeated every 2 weeks

| Drug | Dosage (mg m$^{-2}$) | Time (h) | Infusion duration (h) |
|---|---|---|---|
| PALA[a] | 250 | −24 | 15–20 min |
| Folinic acid | 200 | 0 | 2 h |
| 5-FU | 300–500 | 2 | 10 min (bolus) |
| 5-FU | 300–500 | 2.17 | 22 h |
| Folinic acid | 200 | 24 | 2 h |
| 5-FU | 300–500 | 26 | 10 min (bolus) |
| 5-FU | 300–500 | 26.17 | 22 h |

[a]In 30 patients.

(bolus and infusion): 300 mg m$^{-2}$, three patients; 400 mg m$^{-2}$, 47 patients; 500 mg m$^{-2}$, 45 patients. The results for the 300 and 400 mg m$^{-2}$ dose levels were combined.

Details of sex, age, primary site (colon/rectum), pretreatment performance status and function were also extracted from case notes. Toxicity associated with previous cycles of chemotherapy was recorded at each visit using WHO toxicity criteria.

*Statistical methods*

Survival analyses with covariates were performed using the computer programs BMDP 1L and 2L. Covariates included were: age, co-administration of PALA, 5-FU dosage, performance status (WHO grade), primary site (colon or rectum) and biochemical tests of liver function (ALP, AST and bilirubin). Survival curves were generated using the Kaplan–Meier method (Kaplan & Meier, 1958). Chi-squared tests of association and trend were used as appropriate.

### Results

*Patients*

Ninety-five patients (no previous chemotherapy) received the bolus/infusional 5-FU/folinic acid regimen between March 1991 and May 1992. The median age was 60 (range 27–83) and 55 were male (40 female). Peformance status was generally good: PS 0, 23; PS 1, 55; PS 2, 10; PS 3, 3; PS not recorded 4. In 58 patients the primary disease site was the colon (37 rectum). In 81 patients (85%) sufficient information was available to make them assessable for response. Reasons for inevaluability ($n$ = 14) were as follows: seven patients, no measurable disease; two patients, previous radiotherapy to marker lesion; one patient, toxic death; one patient myocardial infarct after one cycle; one patient, refused further treatment after three cycles and was not reassessed; one patient, died after three cycles and details of cause of death were not available; one patient, developed cerebral metastases after four cycles and systemic disease was not reassessed.

*Response following chemotherapy*

The overall response rate (Table II) was low when assessed 2 months (four cycles) after starting treatment, with only 9 out of 81 evaluable patients (11%, 95% CI = 4–18%) achieving an objective partial response. Disease remained stable in a further 29 patients (36%). The response rates for the different 5-FU dosage regimens and pretreatment performance status are also shown in Table II. Although it was observed that a lower proportion of patients treated at the highest 5-FU dosage (500 mg m$^{-2}$) progressed on treatment, this difference is not statistically significant ($\chi^2_{trend}$ = 0.51, 1 d.f., $P$ = 0.48). Pretreatment performance status had a statistically significant effect on response. If PR + SD and WHO performance status grades 2 and 3 are combined, then patients with a better performance status are more likely to respond ($\chi^2_{trend}$ = 5.78, 1 d.f., $P$ = 0.02). There was no evidence that co-administration of PALA, the presence of visceral metastases, the site of the primary tumour or abnormal liver function tests (ALP, AST, bilirubin) was associated with a change in response rates (data not shown).

*Survival following chemotherapy*

The median survival was 8 months. Seventy-six patients had died, and the median follow-up time in survivors was 17 months (range 9–27). There was a difference in survival between the two 5-FU dosage groups ([log-rank $\chi^2$ = 4.87, 1 d.f., $P$ = 0.03; relative hazard (95% CI) = 0.61 (0.38–0.97]). Adjusting simultaneously for the effects of performance status, age, PALA, liver function (ALP, AST, bilirubin), using a Cox proportional hazards regression model, the effect of 5-FU dosage was even more marked ([relative hazard (95% CI) = 0.38 (0.21–0.70); $\chi^2$ = 10.18, 1 d.f., $P$ = 0.001]) (see Figure 1). Using a stepwise selection procedure, it was found that 5-FU dosage, performance status, bilirubin and the site of primary disease are independently related to survival. The relative hazards (95% CI) are: 500 mg m$^{-2}$ 5-FU dosage, 0.43 (0.26–0.73); performance status 0 or 1, 0.21 (0.10–0.46); bilirubin normal, 0.18 (0.06–0.51); and primary site rectum, 0.59 (0.35–0.97).

**Figure 1** An improvement in median survival was associated with the higher 5-FU dosage: 300–400 mg m$^{-2}$ (...), 5 months; 500 mg m$^{-2}$ (–), 9 months. Relative hazard = 0.38, 95% CI = 0.21–0.70, adjusting simultaneously for the effects of PS, age, PALA, primary site and liver function.

**Table II** Response data

| | Partial response (PR) | Stable disease (SD) | PR + SD | Progression (PD) |
|---|---|---|---|---|
| Overall ($n$ = 81) | 9 (11%) | 29 (36%) | 38 (47%) | 43 (53%) |
| 5-FU dosage (mg m$^{-2}$) | | | | |
| 500 | 5 (12%) | 16 (39%) | 21 (51%) | 20 (49%) |
| 300–400 | 4 (10%) | 13 (32%) | 17 (42%) | 23 (58%) |
| Performance status | | | | |
| 0 | 3 (14%) | 10 (48%) | 13 (62%) | 8 (38%) |
| 1 | 5 (11%) | 17 (37%) | 22 (48%) | 24 (52%) |
| 2 + 3 | 1 (8%) | 1 (8%) | 2 (17%) | 10 (83%) |

Using a Cox proportional hazard model, the effect of symptoms (PS 1) *vs* no symptoms (PS 0) was not significant (relative hazard = 1.17, 95% CI 0.61–2.24, $\chi^2 = 0.24$, 1 d.f., $P = 0.62$) in a survival analysis adjusting simultaneously for age, 5-FU dosage, primary site, PALA and liver function (ALP, AST, bilirubin) (Figure 2).

### *Toxicity associated with chemotherapy*

The regimen was generally well tolerated even at the higher 5-FU dosage (500 mg m$^{-2}$). One patient developed grade 3 diarrhoea in association with grade 4 leucopenia and died. This was considered to be a toxic death. Dosage was reduced as result of diarrhoea in seven patients (5;500–400 mg m$^{-2}$, 2; 400–300 mg m$^{-2}$) and mucositis in one patient (500–400 mg m$^{-2}$. In total, leucopenia was seen in three patients (two grade 4, one grade 2) and 5-FU dosage was reduced (500–400 mg m$^{-2}$) in one patient. Hand-foot syndrome led to dosage reduction in 1 patient (400–300 mg m$^{-2}$). Superficial thrombophlebitis was also noted in a number of patients and rhinitis with blood-stained mucus was described.

There did appear to be an increase in toxicity with the increased 5-FU dosage (Table III). However, these increases were not statistically significant individually. Mucositis (WHO grade 2/3) was seen in seven patients (16%) at 500 mg m$^{-2}$ 5-FU compared with two patients (4%) at 300–400 mg m$^{-2}$; $\chi^2_{trend} = 1.32$, 1 d.f., $P = 0.25$. A similar pattern was seen with diarrhoea ($\chi^2_{trend} = 2.76$, 1 d.f., $P = 0.1$) and nausea and vomiting ($\chi^2_{trend} = 0.57$, 1 d.f., $P = 0.45$). To give a single overall measure, toxicity was summed (mucositis WHO grade + diarrhoea WHO grade + nausea/vomiting WHO grade) for individual patients. Grouping 0 and 1 (minimal overall toxicity), 2 and 3 (moderate) and $\geqslant 4$ (severe), there was a significant increase ($\chi^2_{trend} = 4.44$, 1 d.f., $P = 0.04$) in overall toxicity associated with the higher 5-FU dosage, although the toxicity was generally tolerable.

**Figure 2** In a survival analysis (P2L) adjusting simultaneously for age, 5-FU dosage, primary site, PALA and liver function (ALP, AST, bilirubin) using a Cox proportional hazard model, the effect of symptoms (PS 1) *vs* asymptomatic status (PS 0) was not significant (relative hazard = 1.17, 95% CI 0.61–2.24).

There was no evidence that other variables including performance status and PALA co-administration significantly altered the incidence of toxicity.

### Discussion

This paper has described a restrospective analysis of 95 patients with metastatic colorectal cancer treated using the 5-FU/folinic acid regimen described by De Gramont *et al.* (1988). In agreement with other authors (Johnson *et al.*, 1991; Seymour *et al.*, 1994) we found the regimen to be well tolerated with a low incidence of WHO grade $\geqslant 3$ toxicity. However, although well tolerated, evidence of anti-tumour activity using this regimen was disappointing and did not approach the response rate (54%) or median survival (18 months) which had previously been reported (De Gramont *et al.*, 1988). It is accepted that response rates may vary between studies as a result of interpretation of response criteria and the difficulty in obtaining reproducible bi-dimensional measurements using present imaging techniques. However, in this study in responding patients response duration was short (median 4 months) and median survival was also shorter (8 months) than that previously reported for this regimen.

The multivariate analysis reported in this paper was performed in an attempt to identify factors which may have been responsible for our poor results. All patients included in the analysis had received no prior chemotherapy. The ages of patients studied (median 60, range 27–83) are similar to those in the original report (median 62, range 38–79), and in the multivariate analysis age did not affect survival. Performance status is well recognised as a predictor of outcome in chemotherapy trials. In this analysis the number of patients treated with performance status > 1 was small (13). However performance status (0,1 *vs* 2,3) did have a significant effect on survival and also on response if PR and SD are combined (Table III). In patients with PS = 0 (i.e. asymptomatic) the response rate was 14% (3/21 evaluable) and median survival was 10 months. Therefore PS does not appear to explain the discrepancy between our results and those of De Gramont *et al.* (1988). It was also noted that there was no survival difference in patients who were asymptomatic (PS 0) and those who were symptomatic (PS = 1) (Figure 2).

Three different dosages of 5-FU were administered to patients, although the folinic acid dosage (200 mg m$^{-2}$) and infusion schedule (Table I) remained constant. Therefore, as part of the multivariate analysis, 5-FU dosage was assessed as an independent predictor of response and survival and a trend towards improved survival with increased 5-FU dosage was demonstrated (Figure 1). However, the response rate and survival at the higher 5-FU dosage remains disappointing; (5/41 evaluable = 12%, 95% CI = 2–22%, median survival = 9 months).

The most common grade 3 toxicity was diarrhoea, and yet this occurred in only 6/43 (14%) patients treated at a 5-FU dosage of 500 mg m$^{-2}$. In one patient severe toxicity including grade 4 neutropenia was encountered, and this patient probably died as a result of drug-induced toxicity. Sporadic severe toxicity has been previously reported in patients receiving 5-FU, and this phenomenon has been associated with evidence of reduced activity of the enzyme dihydropyrimidine dehydrogenase (DPD) (Lilenbaum *et al.*, 1991).

**Table III** Toxicity graded using WHO criteria and split by 5-FU dosage (mg m$^{-2}$). Numbers represent worst toxicity at any course

| | *Mucositis* | | *Diarrhoea* | | *Nausea and vomiting* | |
|---|---|---|---|---|---|---|
| *WHO grade* | *400 mg m$^{-2}$ 5-FU* (n = 46)[a] | *500 mg m$^{-2}$ 5-FU* (n = 43) | *400 mg m$^{-2}$ 5-FU* (n = 46)[a] | *500 mg m$^{-2}$ 5-FU* (n = 43) | *400 mg m$^{-2}$ 5-FU* (n = 46)[a] | *500 mg m$^{-2}$ 5-FU* (n = 43) |
| 0 | 33 (72%) | 29 (67%) | 30 (65%) | 22 (51%) | 21 (46%) | 17 (40%) |
| 1 | 11(24%) | 7 (16%) | 9 (20%) | 8 (19%) | 18 (39%) | 17 (40%) |
| 2 | 2 (4%) | 6 (14%) | 6 (13%) | 7 (16%) | 6 (13%) | 7 (16%) |
| 3 | 0 | 1 (2%) | 1 (2%) | 6 (14%) | 1 (2%) | 2 (5%) |

[a]Includes three patients who received 5-FU; 300 mg m$^{-2}$.

but clinical material which might allow the diagnosis of such an enzyme deficiency was not available.

Two toxicities were encountered which had not been anticipated prior to the study. A number of patients noted a superficial phlebitis associated with peripheral infusion sites, and these were occasionally ulcerating. However, phlebitis did not lead to treatment being discontinued in any patients. A number of patients also complained of blood-stained nasal discharge. This was not associated with thrombocytopenia or any other clotting disorder and was thought to be associated with a nasal mucositis similar to that noted in the gastrointestinal tract.

The low incidence of toxicity seen in our analysis and the improved survival with higher 5-FU dosage suggests that higher 5-FU dosage or 5-FU dose intensity may lead to improved anti-tumour activity. The dose intensity of the 5-FU/folinic acid regimen used in the study reported here is 5-FU 1,000 mg $m^{-2}$ $week^{-1}$ and folinic acid 200 mg $m^{-2}$ $week^{-1}$. In comparison, a regimen has been reported in which the 5-FU dose intensity (in combination with folinic acid) has been escalated to 2,600 mg $m^{-2}$ $week^{-1}$ and folinic acid dose intensity to 500 mg $m^{-2}$ $week^{-1}$ (Ardalan *et al.*, 1992). In this study high response rates, albeit in small numbers of patients, have been reported.

We feel that the response rate and survival duration of the order seen in our analysis do not justify the cost of administration in terms of both drug costs and, in our experience in Glasgow, in-patient care. Patients were admitted for two nights every 2 weeks and this is likely to have a negative impact upon quality of life. In one patient this was stated as the cause of significant psychological morbidity and premature cessation of therapy. However, we are sensitive to the fact that this paper describes a retrospective analysis and are aware of the importance of performing carefully monitored prospective analyses of drug regimens. For this reason we are now accruing patients into a formal prospective analysis of this regimen at the higher 5-FU dosage (500 mg $m^{-2}$). In order to limit the number of patients treated using a potentially suboptimal regimen we have initiated a prospective phase II study using a sequential triangular procedure (Whitehead, 1983) for response analysis. On completion of this study we propose to explore the potential for dose/dose intensity escalation which we feel exists. In the meantime we caution against the use of the 5-FU/folinic acid regimen described in this paper as 'uncontrolled standard therapy'.

The authors are grateful to the following consultants for cooperating in the review of patients' case sheets: J.M. Russell, D.J. Kerr (present address Department of Medical Oncology, University of Birmingham), R.P. Symonds, A.G. Robertson, A.N. Harnett, H. Yosef, W.P. Steward, R.D. Jones and F.R. MacBeth.

Dr J. Cassidy and Professor S.B. Kaye are supported by the Cancer Research Campaign. Dr L.S. Murray was supported by the Scottish Hospitals Endowments Research Trust (SHERT, Grant No. 1055).

### References

ADVANCED COLORECTAL CANCER META-ANALYSIS PROJECT (1992). Modulation of fluorouracil by leucovorin in patients with advanced colorectal cancer: evidence in terms of response rate. *J. Clin. Oncol.*, **10**, 896-903.

ARDALAN, B., SRIDHAR, K.S., REDDY, R., BENEDETTO, P., RICHMAN, S., WALDMAN, S., MORRELL, L., FEUN, L. SAVARAJ, N. & LIVINGSTONE, A. (1992). A phase I study of short high dose 5-fluorouracil and high dose leucovorin and low dose phosphonacetyl-L-aspartic acid (PALA) in patients with advanced malignancies. *Int. J. Radiat. Oncol. Biol. Phys.*, **22**, 511-514.

COHEN, S.S., FLAKS, J.G., BARNER, H.D., LOEB, M.R. & LICHTENSTEIN, J. (1958). The mode of action of 5-fluorouracil and its derivatives. *Proc. Natl Acad. Sci. USA*, **44**, 1004.

DE GRAMONT, A., KRULIK, M., CADY, J., LAGADEC, B., MAISANI, J.E., LOISEAU, J.P., GRANGE, J.D., GONZALEZ-CANALI, G., DEMUYNCK, B., LOUVET, C., SEROKA, J. & DEBRAY, J. (1988). High dose folinic acid and 5-fluorouracil bolus and continuous infusion in advanced colorectal cancer. *Eur. J. Cancer*, **24**, 1499-1503.

GREM, J.L., HOTH, D.F., HAMILTON, J.M., KING, S.A. & LEYLAND-JONES, B. (1987). Overview of current status and future direction of clinical trials with 5-fluorouracil in combination with folinic acid. *Cancer Treat. Rep.*, **71**, 1249-1264.

JOHNSON, P.W.M., THOMPSON, P.I., SEYMOUR, M.T., DEASY, N.P. THURAISINGHAM, R.C., SLEVIN, M.L. & WRIGLEY, P.F.M. (1991). A less toxic regimen of 5-fluorouracil and high-dose folinic acid for advanced gastrointestinal adenocarcinomas. *Br. J. Cancer*, **64**, 603-605.

KAPLAN, E.L. & MEIER, P. (1958). Non-parametric estimation from incomplete observations. *J. Am. Stat. Assoc.*, **53**, 457-481.

LILENBAUM, R.C., HARRIS, B.E., DIASIO, R.B., NAES, J. & LYSS, A.P. (1991). Heterozygosity for dihydropyrimidine dehydrogenase (DPD) deficiency may result in life-threatening (Gr4) toxicity from fluorouracil. *Proc. Am. Soc. Clin. Oncol.*, **10**, 120.

MARTIN, D.S., STOLFI, R.L., SAWYER, R.C., SPIEGELMAN, S., CASPER, E.S. & YOUNG, C.W. (1983). Therapeutic utility of utilizing low doses of N-(phosphonacetyl)-L-aspartic acid in combination with 5-fluorouracil: a murine study with clinical relevance. *Cancer Res.*, **43**, 2317-2321.

PETRELLI, N., HERRERA, L., RUSTUM, Y., BURKE, P., CREAVEN, P., STULC, J., EMRICH, L.J. & MITTELMAN, A. (1987). A prospective randomised trial of 5-fluorouracil versus 5-fluorouracil and high-dose leucovorin versus 5-fluorouracil and methotrexate in previously untreated patients with advanced colorectal carcinoma. *J. Clin. Oncol.*, **5**, 10, 1559-1565.

SEYMOUR, M.T., SLEVIN, M., CUNNINGHAM, D., KERR, D., JAMES, R., LEDERMAN, J., PERREN, T., MCADAM, W., DUFFY, A., STENNING, S. & TAYLOR, I. (1994). A randomised trial to assess the addition of interferon-α2a (IFNα) to 5-fluorouracil and leucovorin (LV) in advanced colorectal cancr. *Br. J. Cancer*, **69** (Suppl. XXI), 24.

WHITEHEAD, J. (1983). *The Design and Analysis of Sequential Clinical Trials*. Ellis Horwood: Chichester.